# Tomato Bushy Stunt Virus (TBSV): From a Plant Pathogen to a Multifunctional Biotechnology Platform

**DOI:** 10.3390/v17091268

**Published:** 2025-09-19

**Authors:** Almas Madirov, Nurgul Iksat, Zhaksylyk Masalimov

**Affiliations:** Scientific Laboratory for Plant Biotechnology Named After Rustem Omarov, L. N. Gumilyov Eurasian National University, Astana 010008, Kazakhstan

**Keywords:** Tomato bushy stunt virus, viral vector, biopharming, viral nanoparticles, vaccine platform, drug delivery

## Abstract

Plant viruses have evolved from being viewed exclusively as pathogens into versatile and powerful tools for modern biotechnology. Among them, Tomato bushy stunt virus (TBSV) holds a special place due to its well-studied molecular biology and unique structural properties. This review systematizes the knowledge on TBSV’s dual role as a multifunctional platform. On one hand, we cover its application as a viral vector for the highly efficient expression of recombinant proteins in plants, as well as a tool for functional genomics, including Virus-Induced Gene Silencing (VIGS) and the delivery of CRISPR/Cas9 gene-editing components. On the other hand, we provide a detailed analysis of the use of the stable and monodisperse TBSV virion in nanobiotechnology. Its capsid serves as an ideal scaffold for creating next-generation vaccine candidates, platforms for targeted drug delivery to tumor cells, and as a building block for the programmable self-assembly of complex nanoarchitectures. In conclusion, key challenges limiting the widespread adoption of the platform are discussed, including the genetic instability of vectors and difficulties in scalable purification, along with promising strategies to overcome them.

## 1. Introduction

For decades, plant viruses were seen simply as pathogens, notorious for devastating agricultural crops [1]. However, recent advances in molecular biology and genetic engineering have completely shifted this perspective. Scientists have repurposed these highly efficient biological machines, transforming them from a threat into versatile tools for modern biotechnology [2]. By hijacking a virus’s natural ability to replicate within plant cells, researchers can now address challenges in fields ranging from agronomy to medicine.

The primary advantage of using plant viruses is the speed of expression. Creating genetically stable transgenic plants is a process that can take months or even years [3]. In contrast, virus-based systems can transiently express a target protein transiently in just a few weeks [4]. This rapid approach, often called “molecular farming”, leverages the virus’s ability to replicate and spread systemically, effectively turning the entire host plant into a low-cost, scalable bioreactor [5]. The yields are impressive, often reaching several milligrams of recombinant protein per gram of leaf tissue.

These features have made plant viruses popular platforms for producing therapeutic proteins, antibodies, and industrial enzymes [6]. They have also become essential for functional genomics, allowing researchers to study gene function through a technique known as virus-induced gene silencing (VIGS) [7]. More recently, the uniform and robust structure of viral particles has made them ideal scaffolds in nanobiotechnology for designing vaccines, drug delivery systems, and other nanomaterials [8]. As a result, plant viruses now stand as a powerful and flexible platform, driving innovation in “green” biotechnology.

## 2. TBSV

Among the many viral systems adapted for biotechnology, Tomato bushy stunt virus (TBSV) holds a special place. It was first described in 1935 infecting tomatoes, and later it was found in peppers, cherry, pear, statice, and other plant species [9,10,11,12]. Symptoms vary depending on the host plant and can be mild (e.g., growth stunting and systemic mosaic) or severe, such as lethal systemic necrosis in *Nicotiana benthamiana*. Natural transmission occurs primarily through the soil, and less often through water, infected plant residues, or tools; known insect vectors are absent [9]. Concurrently, the experimental host range of TBSV is very wide and covers more than 120 plant species from 20 families, which makes it a universal tool in laboratory research [9].

**Figure 1:**
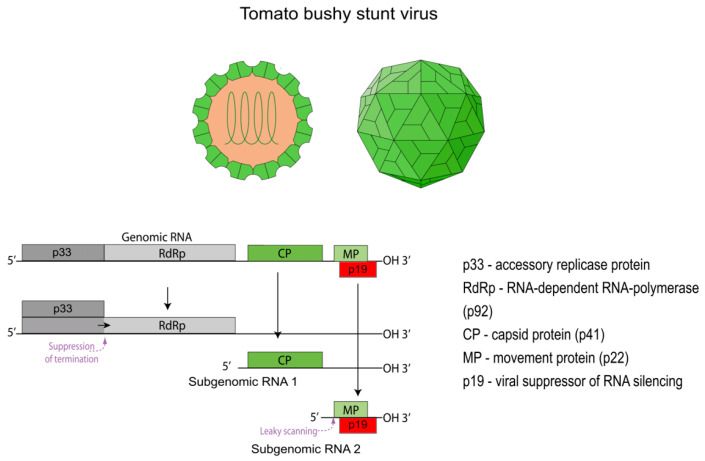
Schematic representation of the virion and genome organization of Tomato bushy stunt virus (TBSV). The figure includes two visualizations of the icosahedral virion structure and a detailed map of the TBSV genome. The genome map illustrates the expression strategy for the key viral proteins, including the synthesis of two subgenomic RNAs. Adapted from the ViralZone illustrations, which are licensed under a CC BY 4.0 license [19].

As a typical representative of the *Tombusviridae* family and the *Tombusvirus* genus, TBSV has an icosahedral virion with a diameter of 30 nm [13]. The TBSV life cycle within a host cell begins with the penetration of the virion and the subsequent release of its genomic RNA. This uncoating process is thought to be triggered by the removal of Ca^2+^ ions from the capsid within the cellular environment, which leads to a reversible “swelling” of the particle and the release of its RNA [14,15]. The released genome is a single-stranded, positive-sense RNA (+ssRNA) of approximately 4.8 kb [16]. A distinctive feature of the TBSV genome is the absence of a 5′ cap structure and a 3′ poly(A) tail. The genome contains five open reading frames (ORFs) that encode the key viral proteins (Figure 1) [16]. To express these proteins, the virus employs a complex strategy involving translation from both the genomic RNA and two subgenomic RNAs (sgRNA 1 and sgRNA 2) [17]. Despite lacking the canonical structures for translation initiation in eukaryotes, all viral proteins are synthesized efficiently. This is achieved through a cap-independent translation mechanism, which relies on complex RNA secondary structures and a long-distance RNA-RNA interaction between the 5′ and 3′ ends of the genome [18].

### 2.1. The Replicase Complex: p33 and p92

The first two proteins, p33 and p92, are translated directly from the genomic RNA [20]. The p92 protein is a C-terminal extension of p33; its synthesis occurs via ribosomal read-through of the p33 stop codon [21]. This mechanism ensures that the ratio of p33 to p92 is approximately 20 to 1, which is critically important for proper replication [21]. These two proteins form the viral replicase complex. The larger p92 protein is the RNA-dependent RNA polymerase (RdRp), responsible for synthesizing new viral RNAs, while p33 acts as an auxiliary replication protein. Its main function is to recruit viral RNA and host factors to assemble the viral replication organelle (VRO) [22]. These VROs are formed by the invagination of the peroxisome membrane and provide a protected microenvironment for viral replication. It is here that the replicase complex synthesizes not only new copies of the genomic RNA but also the two sgRNAs, using the negative-sense genomic RNA as a template [22].

### 2.2. Accessory Proteins: p22 and p19

The remaining two proteins, p22 and p19, are expressed from sgRNA 2 and are encoded by ORF4 and ORF5, respectively [23,24]. The synthesis of p22 and p19 proteins is regulated by a leaky scanning mechanism. The start codon of ORF4, encoding p22, is situated within a weak Kozak sequence context, allowing the ribosome to bypass it and initiate translation from the downstream start codon of ORF5. Consequently, the p19 protein is synthesized in significantly larger quantities than p22. Weak expression of the p22 protein is explained by its function. It is a membrane-associated and RNA-binding protein that is only needed for the movement of the virus between adjacent plant cells through plasmodesmata [25]. At the same time, the p19 protein plays a key role in the virus’s pathogenicity, being a powerful viral suppressor of RNA silencing [26,27,28,29]. The special structure of this protein is aimed at binding double-stranded short RNAs (siRNAs), mostly with a length of 21 nucleotides [30]. Such siRNAs are formed by the activity of Dicer-like (DCL) proteins when foreign RNA appears in the cell. They are then incorporated into the RNA-induced silencing complex (RISC), which executes the targeted degradation of exogenous RNAs [31]. By sequestering the siRNA, the p19 protein effectively neutralizes the main antiviral defense mechanism of the plant, ensuring active replication of the virus [32].

### 2.3. Virion Structure and Assembly

The final viral structure, the virion, exhibits exceptional stability at high temperatures and in the presence of various detergents [33]. The capsid is formed from 180 copies of the coat protein (CP), designated p41 (41 kDa), which is encoded by ORF3 and synthesized from sgRNA 1 [34].

The p41 protein has a three-domain structure. The N-terminal RNA-binding domain (R-domain) is located on the interior of the capsid and interacts with the viral RNA genome. The shell domain (S-domain) forms the continuous, rigid icosahedral shell. Finally, the C-terminal protruding domain (P-domain) extends from the virion’s outer surface and has a flexible structure, connected to the S-domain by a short hinge region [34].

The final virion is assembled from 180 copies of the p41 protein, which are organized with T = 3 quasi-equivalent symmetry. The protein subunits adopt one of three slightly different conformations (A, B, or C) to form pentamers and hexamers on the capsid surface. The stability and integrity of the virion are further reinforced by calcium ions (Ca^2+^), which are located at the interfaces between protein subunits and act as structural clamps [14]. While not required for the assembly itself, these ions are critical for the mechanical strength of the assembled particle. The final step of the viral life cycle, encapsidation, involves the packaging of a newly synthesized genomic RNA molecule within the self-assembling shell of 180 p41 subunits, forming a mature, infectious virion ready to spread.

Due to its unique characteristics, TBSV has become one of the most studied and robust model systems for researching fundamental aspects of virus-plant interactions, including the mechanisms of RNA replication, virus transport in the plant, and plant antiviral mechanisms. Its relatively small and compact genome (~4.8 kb), coupled with the development of infectious cDNA clones and a unique yeast-based replication system (*Saccharomyces cerevisiae*), has facilitated deep mechanistic studies of viral replication and the hijacking of host cell machinery [35]. In addition, structural studies of the capsid and suppressor proteins have elucidated the physicochemical basis of their functions. This deep molecular understanding directly contributed to its transformation from a plant pathogen into a multifunctional biotechnology platform.

## 3. TBSV as a Multifunctional Vector

One of the first and most significant applications of TBSV in biotechnology is its conversion into an expression vector system. Creating these vectors primarily involves replacing the CP gene, which is dispensable for viral replication and local movement in many host plants [36]. The coding sequence of a gene of interest (GOI) is inserted in its place. During viral infection, the vector RNA replicates to high levels, leading to the overexpression of the target protein. This platform is used for a wide range of applications, from producing recombinant proteins (biopharming) to functional genomics studies using VIGS and delivering CRISPR/Cas9 components.

### 3.1. Vector Construction

**Figure 2:**
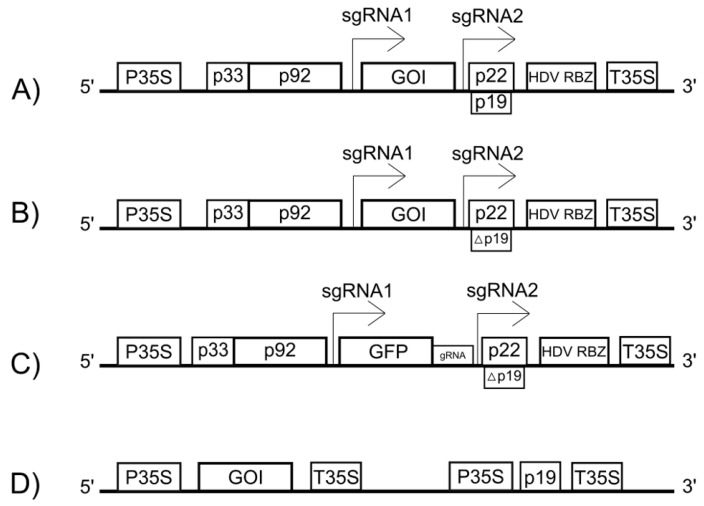
Schematic representation of the T-DNA region of binary vectors used for expressing TBSV-based constructs. Typically, the viral cDNA is placed under the control of the Cauliflower mosaic virus 35S promoter (P35S) and is followed by the Hepatitis Delta Virus ribozyme (HDV RBZ) and a CaMV 35S transcription terminator (T35S). Bent arrows indicate the transcription initiation sites for the subgenomic RNAs (sgRNA1 and sgRNA2). (**A**) A standard expression vector. (**B**) A vector for Virus-Induced Gene Silencing (VIGS). (**C**) A vector for the CRISPR/Cas9 system, which includes a green fluorescent protein (GFP) reporter gene. (**D**) A co-expression scheme where the p19 gene is supplied from a separate vector. Abbreviations: GOI, gene of interest; gRNA, guide RNA; Δ—knock out.

Modern TBSV-based vectors are plasmids designed for delivery into plant cells, most commonly via agroinfiltration. In these plasmids, a full-length cDNA copy of the TBSV genome is placed under the control of a strong constitutive promoter, such as the Cauliflower mosaic virus (CaMV) 35S promoter, which drives transcription in the plant cell nucleus (Figure 2) [37].

A critical aspect for successful vector replication is the precise formation of the native viral 3′ end. Transcription initiated by the 35S promoter requires a transcription termination and polyadenylation signal (e.g., T35S) to function correctly, which adds a poly(A) tail to the transcript [38]. However, the native TBSV RNA lacks a poly(A) tail and requires a specific 3′ terminus to be recognized by the viral replicase. This challenge is overcome by inserting a self-cleaving ribozyme sequence (e.g., from Hepatitis Delta Virus, HDV RBZ) immediately downstream of the viral genome but upstream of the T35S terminator (Figure 2A) [39]. Inside the nucleus, a long precursor RNA is transcribed. The ribozyme then self-cleaves, precisely removing itself and all downstream sequences, including the poly(A) tail. This process generates a viral RNA with an authentic 3′ end, which is then exported to the cytoplasm to initiate replication.

The expression of the GOI, inserted in place of p41 (Figure 2A), is driven by the highly active subgenomic promoter that normally controls the synthesis of the coat protein. During replication, the viral polymerase produces a large number of sgRNA1 copies, which now serve as the mRNA for the GOI, leading to its high-level accumulation in the cytoplasm.

The vector designs described above have been successfully employed for numerous applications in plant biotechnology, the key examples of which are summarized in Table 1.

### 3.2. TBSV in Functional Genomic Studies

TBSV-based vectors are powerful tools for fundamental scientific research (Table 1). One key technique is VIGS. This approach utilizes a vector based on a mutant TBSV with an inactivated p19 silencing suppressor, into which a small fragment of a host target gene is inserted (Figure 2B) [44]. The replication of this vector in the plant cell leads to the accumulation of siRNAs directed against the target gene’s mRNA, resulting in a gene knockdown that allows researchers to study its function based on the resulting phenotype.

Additionally, the TBSV vector system can be used to deliver components of the CRISPR/Cas9 gene-editing system into plant cells (Figure 2C) [45]. Specifically, a TBSV vector can be engineered to carry and express single guide RNAs (gRNAs) designed to target specific host genes. When this gRNA-expressing virus is delivered together with a separate vector that expresses the Cas9 nuclease, it provides rapid and efficient gene editing in planta without the need for stable transformation.

### 3.3. Biopharming

One of the main applications for TBSV-based vectors is biopharming—the production of clinically and commercially important proteins in plants. The platform is used for both research purposes, such as expressing reporter genes like GFP and β-glucuronidase (GUS), and for producing therapeutic proteins [43,44,46]. Thanks to the high replication efficiency of the viral vector, the yield of recombinant protein can reach significant levels, as was the case for human granulocyte colony-stimulating factor (G-CSF) (Table 1) [42]. Furthermore, these systems are successfully used to produce antigens for vaccines and diagnostics. TBSV vectors have been used to express proteins such as the HIV-1 nucleocapsid protein p24 and the bovine leukemia virus (BLV) envelope glycoprotein gp51 (Table 1) [40,41]. It has also been shown that two different proteins can be expressed simultaneously in the same plant cells by co-inoculation with vectors based on TBSV and Tobacco mosaic virus (TMV), which opens up possibilities for studying protein–protein interactions [47].

However, besides the virus itself, the p19 protein is also used separately to increase the expression of transgenic proteins (Figure 2D). Its ability to suppress RNA interference significantly prolongs the transient expression of target proteins [48]. This can be achieved by using a vector containing the p19 gene under the control of a strong promoter, such as the CaMV 35S promoter [49,50].

## 4. Applications of the TBSV Virion in Nanobiotechnology

In addition to the use of the TBSV genome for creating vectors, its capsid is equally compelling. A thorough understanding of the physicochemical properties of the TBSV virion has opened up new avenues for its application in nanobiotechnology [14]. The attractiveness of TBSV for this field stems from several fundamental characteristics.

First, TBSV nanoparticles can be produced quickly and inexpensively on a large scale. Host plants, particularly *N. benthamiana*, serve as the main system for producing full-fledged virions, while insect cell-based systems are used to produce empty virus-like particles (VLPs) [27,51]. Second, TBSV virions themselves are ideal nanostructures: they are monodisperse and self-assemble from 180 identical copies of the coat protein [52]. This makes them an ideal scaffold for the polyvalent display of foreign antigens on the surface [53].

Finally, a key advantage for medical use is the high degree of biocompatibility and safety [54]. As a plant virus, TBSV is unable to replicate in mammalian cells, and humans lack pre-existing immunity to it. Studies have confirmed that TBSV nanoparticles are non-toxic and non-teratogenic [55].

Key strategies for the functionalization of TBSV nanoparticles based on these properties are summarized in Table 2.

### 4.1. Virion Modifications

**Figure 3:**
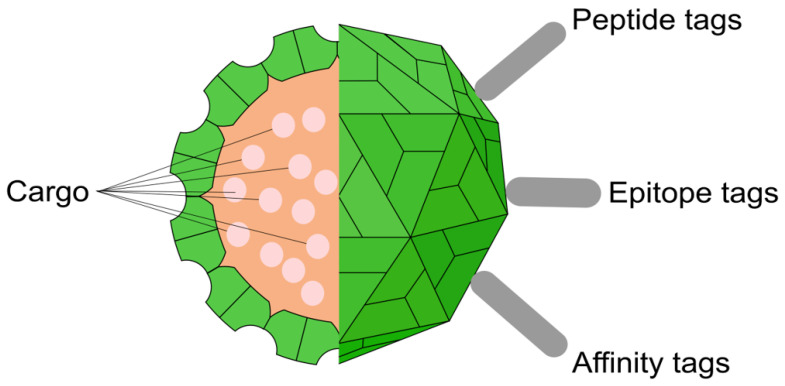
Functionalization strategies for TBSV nanoparticles. This schematic illustrates the main strategies for modifying TBSV nanoparticles for biotechnological applications. The outer surface can be functionalized by attaching various molecules, such as targeting peptide tags, antigenic epitope tags, or affinity tags, to the protruding domains of the coat protein. The internal cavity can be utilized as a nanocontainer to encapsulate therapeutic or imaging agents, referred to as cargo. Adapted from the ViralZone illustrations, which are licensed under a CC BY 4.0 license [19].

To confer new functions to TBSV nanoparticles, various modification strategies are used, affecting both the outer surface and the inner cavity (Figure 3). The production system for these modified particles depends on the desired final product. For creating infectious, chimeric virions that can replicate and spread, the modifications are introduced into the full-length viral vector, which is then expressed in plants. In contrast, for producing non-infectious, empty VLPs, only the modified coat protein gene is expressed under a strong constitutive promoter in a heterologous system, such as bacteria, yeast, or insect cells. Crucially, studies have shown that such VLPs produced in heterologous systems do not contain the viral genome; instead, they non-specifically encapsidate a mixture of cellular and degraded mRNAs from the host cell [59].

The most common approach for generating chimeric virions is the genetic modification of the capsid surface (Table 2). This process is carried out at the DNA level by engineering the viral cDNA clone within a plasmid. The sequence encoding a target peptide is inserted into the p41 gene immediately upstream of its stop codon. This modified plasmid is then delivered into plants, where during viral infection, a chimeric coat protein is synthesized and self-assembles in vivo into virions that display the desired peptide on their surface.

In addition to genetic engineering, the outer surface of the virion can be modified by chemical conjugation (Table 2). For example, fluorescent dyes can be covalently attached to available cysteine residues on the capsid surface, and biotinylation has been performed on specially introduced lysine residues [52].

Finally, the internal cavity of the virion can be used as a nanocontainer for therapeutic agents (Table 2). Loading is achieved via a reversible capsid “swelling” process, which is induced by the removal of Ca^2+^ ions with a chelating agent like EDTA in an alkaline buffer. This temporarily opens pores in the capsid, allowing drug molecules, such as doxorubicin, to diffuse inside [56]. Subsequent restoration of an acidic pH and the addition of Ca^2+^ ions cause the pores to “close,” securely trapping the cargo.

### 4.2. From Antigen to Drug: The Therapeutic Potential of TBSV

The application of TBSV-based nanoparticles in biomedicine began with their utilization as a platform for vaccine development. Engineered TBSV virus-like particles, displaying 180 copies of a 16-amino acid epitope from the ricin toxin on their surface, elicited a strong immune response upon injection into mice [51]. This resulted in the production of antibodies that could recognize the native toxin. This work demonstrated that TBSV is a versatile platform for the polyvalent display of antigenic epitopes and for vaccine design.

Later, the therapeutic potential of TBSV was studied for treating autoimmune diseases. TBSV nanoparticles displaying immunodominant peptides related to rheumatoid arthritis (pLIP1 and pFADK2) demonstrated the ability to alleviate disease symptoms in a mouse model of collagen-induced arthritis [57]. In this mechanism, the viral particle acted not only as a scaffold for presenting peptides, but also as an adjuvant, enhancing their regulatory activity and stimulating the production of the anti-inflammatory cytokine IL-10. Concurrently, another approach utilized a structural element of TBSV, the β-annulus peptide, as a nanoscaffold to develop a vaccine against Seneca Valley virus A (SVA) in pigs [60]. This 24-mer nanoscaffold, conjugated to SVA antigens, elicited a potent humoral and cellular immune response in the animals. It conferred 80% protection against viral challenge, a level comparable to the efficacy of an inactivated vaccine.

In recent years, the main focus has shifted to the development of drug delivery systems based on TBSV for treating cancer, specifically medulloblastoma, a malignant brain tumor. To achieve this, targeting peptides (e.g., CooP or RPAR) were genetically fused to the surface of TBSV nanoparticles [56,61,62]. These peptides are able to recognize specific receptors on the surface of cancer cells, such as FABP3 and neuropilin-1. Studies have shown that such functionalized particles are specifically absorbed by medulloblastoma cells in vitro. Loading these particles with doxorubicin facilitated the death of 90% of cancer cells at a drug concentration fivefold lower than that of free doxorubicin. Subsequent preclinical studies in mouse models confirmed these results in vivo. Multiple injections of doxorubicin loaded into TBSV-CooP nanoparticles effectively suppressed the growth of tumor foci at early stages. This treatment did not induce the systemic toxicity (e.g., weight loss) observed with the administration of the free drug. These studies ultimately confirmed that TBSV is an effective and safe platform for the targeted delivery of therapeutic agents to brain tumors, opening new perspectives in cancer treatment.

### 4.3. Programmable Assembly: From Monolayers to Complex Nanoarchitectures

While TBSV nanoparticles are viewed primarily as individual functional units in biomedical applications, their value in the field of nanomaterials lies in their ability to act as building blocks for creating larger, more complex systems. The goal of creating such ordered nanoarchitectures is to develop novel functional materials for applications in areas like biosensing, biocatalysis, and nanoelectronics. A key challenge in this field is controlling two processes: first, the formation of a uniform monolayer on a substrate, and second, its subsequent use for the layer-by-layer assembly of more complex structures.

Initially, non-specific electrostatic interactions between the virion and the substrate surface were used to form 2D layers [63,64,65,66]. It was found that the most ordered monolayers form under conditions of weak mutual repulsion, allowing the particles to organize freely under the influence of capillary forces during drying. The key factor for controlling this process was the relationship between the isoelectric points of the virus and the substrate; maximum coverage was achieved when the solution’s pH was between the IEP values, creating conditions for their mutual attraction [67,68]. However, the main drawback of this approach was the low stability of the resulting layers, which were partially washed away during subsequent manipulations, making it impossible to create reliable multilayer systems.

The stability problem was solved by transitioning to a “key-lock” approach based on strong and specific molecular interactions [58,68]. A prime example is the Strep-Tactin/Strep-Tag II system, where a silicon surface is chemically modified with the Strep-Tactin protein, which then strongly and specifically binds virions carrying the Strep-Tag II tag. This approach allows for the formation of extremely stable monolayers that can even withstand ultrasound treatment. It was this reliable foundation that enabled the creation of complex 3D architectures through sequential layer deposition, as demonstrated by the assembly of a robust four-layer structure. Alongside the creation of continuous layers, methods for patterning them have also been developed, such as “carving” patterns with a focused ion beam or directly “drawing” viral structures with fluidic force microscopy [67].

## 5. Critical Analysis: Problems and Future Perspectives

Despite significant advancements, realizing the full potential of the TBSV platform requires overcoming several key challenges. One problem is the genetic instability of foreign genes inserted into the viral vector, as they can be deleted during the replication process. Furthermore, the icosahedral structure of the virion imposes strict limitations on the size of the RNA that can be packaged, which limits the vector’s cargo capacity. In addition, significant limitations are related to the host plant: the p19 protein, while key for high expression levels, can cause severe lethal necrosis in the most common experimental host, *Nicotiana benthamiana*, particularly under high viral replication pressure.

Additional challenges arise when transitioning to clinical applications. These include the potential immunogenicity of the viral particles upon administration, as well as the complexities of downstream processing and purification. It is here that an important distinction must be made: while the upstream production of viral biomass in plants is indeed inexpensive and scalable, the downstream purification required to achieve the purity and safety standards for clinical use is complex and costly. Traditional purification methods, such as ultracentrifugation, are time-consuming and do not yield material in preparative quantities. Viral particles are large, complex structures, which complicates standard chromatography. The final product must be thoroughly purified to remove plant-specific contaminants such as alkaloids and phenolic compounds, as well as bacterial endotoxins. Compliance with strict regulatory standards for purity is a mandatory requirement for any biomedical product.

To overcome these problems, active interdisciplinary research is being conducted, opening new perspectives for the TBSV platform. For instance, the necrosis problem is being addressed by using an alternative host, *Nicotiana excelsiana*, in which TBSV-based vectors cause asymptomatic infection while still achieving high protein expression levels [69]. In the field of nanomedicine, future work will focus on optimizing drug delivery, particularly on overcoming the aggregation of modified nanoparticles and enhancing their penetration across the blood–brain barrier, as well as on expanding the vaccine portfolio to combat a wide range of pathogens. Furthermore, new opportunities are emerging for engineering the nanocontainers themselves; it has been shown that even minor deletions in the N-terminal domain of the coat protein can alter the geometry of capsid assembly, making it possible to create particles of different sizes and symmetries (e.g., T = 1 and T = 3) [59]. A promising direction is also the creation of advanced tools for genetic engineering; future developments may include packaging both gRNA and Cas9 mRNA into a single virus-like particle.

While the CP-replacement strategy remains the dominant and highly effective approach for localized gene expression, the development of stable systemic vectors remains a key challenge. Overcoming the inherent genetic instability of larger inserts in the TBSV genome could unlock new applications. Future research could explore advanced strategies to co-express a foreign gene while retaining the essential coat protein. One potential approach could involve expressing the GOI and p41 as a single polyprotein, linked by a self-cleaving 2A peptide or a specific protease cleavage site. This would ensure stoichiometric production of both proteins from a single sgRNA. Another ambitious strategy could be to engineer the TBSV genome to produce a third, artificial sgRNA, which would require the identification and insertion of a novel subgenomic promoter. Although technically challenging, such next-generation vectors would combine high expression levels with the ability to spread systemically, significantly expanding the utility of the TBSV platform.

Addressing the purification challenge is crucial for clinical applications. One promising solution is the development of rapid methods like one-step hydroxyapatite chromatography [70]. This method has been shown to efficiently separate viral particles from host plant proteins, yielding homogenous and biologically active material suitable for diagnostics and as nanocarriers for vaccines and drugs; furthermore, the method is economical, rapid, and scalable.

## 6. Conclusions

TBSV represents a prime example of how in-depth fundamental research into a plant pathogen can lead to the development of a multifaceted and powerful biotechnology platform. Its well-characterized molecular biology has served as the foundation for innovative applications in medicine and agriculture. As a nanoplatform, TBSV offers a compelling combination of safety, biocompatibility, low-cost production, and high scalability in plants. In the field of plant genetic engineering, TBSV has established itself as a versatile tool for protein expression, VIGS, and even the delivery of CRISPR/Cas9 components.

Despite these advancements, significant challenges remain that must be addressed. These include the genetic instability of vectors, cargo capacity limitations, immunogenicity, and most importantly, the complexities of downstream processing and purification required to meet stringent regulatory standards. The future success of TBSV-based biotechnology will depend on interdisciplinary efforts aimed at addressing these challenges. Nevertheless, TBSV has already firmly established its niche as a unique and promising platform, demonstrating how viruses can be transformed into valuable allies in solving the complex challenges of modern biotechnology.

## Figures and Tables

**Table 1:** Summary of biotechnological applications of TBSV-based vectors. The table provides representative examples demonstrating the versatility of the TBSV-based vector platform. For each major application area, a specific GOI, the typical host plant, and the resulting outcome are listed.

Application	GOI	Host Plant	Result/Outcome	Reference
Biopharming	HIV-1 nucleocapsid protein p24	*N.benthamiana*	Production and accumulation of the recombinant antigen	[40]
	Bovine leukemia virus glycoprotein gp51	*N. benthamiana*	Production and accumulation of the recombinant antigen	[41]
	Human G-CSF	*N. benthamiana*	Yields up to 250 mg/kg of leaf mass	[42]
Reporter Genes	GFP	*N. benthamiana*	rRNA gene promoter analysis	[43]
VIGS	GFP and inactive p19	*N. benthamiana*	Vector with defective p19 can be used to screen VIGS	[44]
CRISPR/Cas9	Guide RNA (gRNA)	*N. benthamiana*	Effective delivery and expression of gRNA for targeted mutagenesis	[45]

**Table 2:** Examples of TBSV nanoparticle functionalization for nanobiotechnology and medicine. The table summarizes key strategies for the modification of TBSV virions. Examples include genetic fusion and chemical conjugation for surface modification, as well as the encapsulation of functional molecules within the internal cavity for targeted delivery.

Modification Type	Molecule Attached/Cargo	Purpose/Application	Key Finding	Reference
Genetic Fusion (Surface)	Ricin toxin epitope	Vaccine candidate design	Elicited antibodies that recognize the native toxin	[51]
	RPAR, CooP peptides	Targeted drug delivery to tumors	Specific uptake by medulloblastoma cells in vitro	[56]
	Rheumatoid arthritis peptides	Treatment of autoimmune disease	Alleviated arthritis symptoms and stimulated anti-inflammatory cytokine IL-10	[57]
	Strep-Tag II affinity tag	Creation of ordered nanostructures	Formation of highly stable monolayers that withstand ultrasound treatment	[58]
Chemical Conjugation (Surface)	Fluorescent dyes, biotin	Labeling and detection	Successful surface modification without disrupting virion structure	[52]
Internal Loading (Cavity)	Doxorubicin	Delivery of a chemotherapy agent	Achieved 90% cancer cell death at a 5-fold lower dose vs. free drug	[56]

## Data Availability

No new data were created or analyzed in this study.

## References

[1] Tatineni S., Hein G.L. (2023). Plant Viruses of Agricultural Importance: Current and Future Perspectives of Virus Disease Management Strategies. Phytopathology.

[2] Venkataraman S., Hefferon K. (2021). Application of Plant Viruses in Biotechnology, Medicine, and Human Health. Viruses.

[3] Ullrich K.K., Hiss M., Rensing S.A. (2015). Means to optimize protein expression in transgenic plants. Curr. Opin. Biotechnol..

[4] eyret H., Lomonossoff G.P. (2015). When plant virology met *Agrobacterium*: The rise of the deconstructed clones. Plant Biotechnol. J..

[5] Zahmanova G., Aljabali A.A., Takova K., Toneva V., Tambuwala M.M., Andonov A.P., Lukov G.L., Minkov I. (2023). The Plant Viruses and Molecular Farming: How Beneficial They Might Be for Human and Animal Health?. Int. J. Mol. Sci..

[6] Debnath S., Seth D., Pramanik S., Adhikari S., Mondal P., Sherpa D., Sen D., Mukherjee D., Bhattacharjee R., Mukerjee N. (2022). A comprehensive review and meta-analysis of recent advances in biotechnology for plant virus research and significant accomplishments in human health and the pharmaceutical industry. Biotechnol. Genet. Eng. Rev..

[7] Rössner C., Lotz D., Becker A. (2022). VIGS Goes Viral: How VIGS Transforms Our Understanding of Plant Science. Annu. Rev. Plant Biol..

[8] Alemzadeh E., Dehshahri A., Izadpanah K., Ahmadi F. (2018). Plant virus nanoparticles: Novel and robust nanocarriers for drug delivery and imaging. Colloids Surf. B Biointerfaces.

[9] Yamamura Y., Scholthof H.B. (2005). *Tomato bushy stunt virus*: A resilient model system to study virus–plant interactions. Mol. Plant Pathol..

[10] Smith K.M. (1935). A New Virus Disease of Tomatoes. Nature.

[11] Havelda Z., Szittya G., Burgyán J. (1998). Characterization of the Molecular Mechanism of Defective Interfering RNA-Mediated Symptom Attenuation in Tombusvirus-Infected Plants. J. Virol..

[12] Hillman B.I., Carrington J.C., Morris T.J. (1987). A defective interfering RNA that contains a mosaic of a plant virus genome. Cell.

[13] Tombusviridae|ICTV. https://ictv.global/report_9th/RNApos/Tombusviridae.

[14] Llauró A., Coppari E., Imperatori F., Bizzarri A.R., Castón J.R., Santi L., Cannistraro S., de Pablo P.J. (2015). Calcium Ions Modulate the Mechanics of Tomato Bushy Stunt Virus. Biophys. J..

[15] Krüse J., Krüse K., Witz J., Chauvin C., Jacrot B., Tardieu A. (1982). Divalent ion-dependent reversible swelling of tomato bushy stunt virus and organization of the expanded virion. J. Mol. Biol..

[16] Madirov A., Yermukhambetova R., Masalimov Z. (2023). Exploring the diversity and evolution of tombus-like viruses: Phylogenetic analysis, recombination events, and suppressor protein homologs. Arch. Virol..

[17] Sztuba-Solińska J., Stollar V., Bujarski J.J. (2011). Subgenomic messenger RNAs: Mastering regulation of (+)-strand RNA virus life cycle. Virology.

[18] Fabian M.R., White K.A. (2004). 5′-3′ RNA-RNA Interaction Facilitates Cap- and Poly(A) Tail-independent Translation of Tomato Bushy Stunt Virus mRNA. J. Biol. Chem..

[19] Hulo C., de Castro E., Masson P., Bougueleret L., Bairoch A., Xenarios I., Le Mercier P. (2010). ViralZone: A knowledge resource to understand virus diversity. Nucleic Acids Res..

[20] Gursinsky T., Schulz B., Behrens S.-E. (2009). Replication of Tomato bushy stunt virus RNA in a plant in vitro system. Virology.

[21] Scholthof K.-B.G., Scholthof H.B., Jackson A. (1995). The Tomato Bushy Stunt Virus Replicase Proteins Are Coordinately Expressed and Membrane Associated. Virology.

[22] Nagy P.D., Feng Z. (2021). Tombusviruses orchestrate the host endomembrane system to create elaborate membranous replication organelles. Curr. Opin. Virol..

[23] Scholthof H.B., Desvoyes B., Kuecker J., Whitehead E. (1999). Biological Activity of Two Tombusvirus Proteins Translated from Nested Genes Is Influenced by Dosage Control via Context-Dependent Leaky Scanning. Mol. Plant-Microbe Interact..

[24] Scholthof H.B., Scholthof K.-B.G., Kikkert M., Jackson A. (1995). Tomato Bushy Stunt Virus Spread Is Regulated by Two Nested Genes That Function in Cell-to-Cell Movement and Host-Dependent Systemic Invasion. Virology.

[25] Chu M., Park J.-W., Scholthof H.B. (1999). Separate Regions on the Tomato Bushy Stunt Virus p22 Protein Mediate Cell-to-Cell Movement versus Elicitation of Effective Resistance Responses. Mol. Plant-Microbe Interact..

[26] Omarov R., Sparks K., Smith L., Zindovic J., Scholthof H.B. (2006). Biological Relevance of a Stable Biochemical Interaction between the Tombusvirus-Encoded P19 and Short Interfering RNAs. J. Virol..

[27] Qu F., Morris T.J. (2002). Efficient Infection of *Nicotiana benthamiana* by *Tomato bushy stunt virus* Is Facilitated by the Coat Protein and Maintained by p19 Through Suppression of Gene Silencing. Mol. Plant-Microbe Interact..

[28] Scholthof H.B. (2006). The Tombusvirus-encoded P19: From irrelevance to elegance. Nat. Rev. Microbiol..

[29] Turina M., Omarov R., Murphy J.F., Bazaldua-Hernandez C., Desvoyes B., Scholthof H.B. (2002). A newly identified role for *Tomato bushy stunt virus* P19 in short distance spread. Mol. Plant Pathol..

[30] Dana H., Chalbatani G.M., Mahmoodzadeh H., Karimloo R., Rezaiean O., Moradzadeh A., Mehmandoost N., Moazzen F., Mazraeh A., Marmari V. (2017). Molecular Mechanisms and Biological Functions of siRNA. Int. J. Biomed. Sci..

[31] Scholthof H.B. (2007). Heterologous Expression of Viral RNA Interference Suppressors: RISC Management. Plant Physiol..

[32] Hsieh Y.-C., Omarov R.T., Scholthof H.B. (2009). Diverse and Newly Recognized Effects Associated with Short Interfering RNA Binding Site Modifications on the *Tomato Bushy Stunt Virus* P19 Silencing Suppressor. J. Virol..

[33] Allen W.R., Davidson T.R. (1967). TOMATO BUSHY STUNT VIRUS FROM PRUNUS AVIUM L.: I. FIELD STUDIES AND VIRUS CHARACTERIZATION. Can. J. Bot..

[34] Olson A., Bricogne G., Harrison S. (1983). Structure of tomato bushy stunt virus IV. J. Mol. Biol..

[35] Pogany J., Nagy P.D. (2008). Authentic Replication and Recombination of *Tomato Bushy Stunt Virus* RNA in a Cell-Free Extract from Yeast. J. Virol..

[36] Scholthof H.B. (1993). The Capsid Protein Gene of Tomato Bushy Stunt Virus Is Dispensable for Systemic Movement and Can Be Replaced for Localized Expression of Foreign Genes. Mol. Plant-Microbe Interact..

[37] Qiu W., Scholthof H.B. (2007). Using Vectors Derived from Tomato Bushy Stunt Virus (TBSV) and TBSV Defective Interfering RNAs (DIs). Curr. Protoc. Microbiol..

[38] Azzoni A.R., Ribeiro S.C., Monteiro G.A., Prazeres D.M. (2007). The impact of polyadenylation signals on plasmid nuclease-resistance and transgene expression. J. Gene Med..

[39] Been M.D., Wickham G.S. (1997). Self-Cleaving Ribozymes of Hepatitis Delta Virus RNA. Eur. J. Biochem..

[40] Zhang G., Leung C., Murdin L., Rovinski B., White K.A. (2000). In planta expression of HIV-1 p24 protein using and RNA plant virus-based expression vector. Mol. Biotechnol..

[41] Zhumabek A.T., Abeuova L.S., Mukhametzhanov N.S., Scholthof H.B., Ramankulov Y.M., Manabayeva S.A. (2018). Transient expression of a bovine leukemia virus envelope glycoprotein in plants by a recombinant TBSV vector. J. Virol. Methods.

[42] Abeuova L., Scholthof H., Ramankulov E., Manabayeva S. (2015). Transient Expression of Human G-Csf In Nicotiana benthamiana Plants Using a Tomato Bushy Stunt Virus–Based Vector. Biotechnol. Theory Pr..

[43] Xu L., Li Z., Wang S. (2021). Development of a Virus-Based Reporter System for Functional Analysis of Plant rRNA Gene Promoter. Front. Microbiol..

[44] Shamekova M., Mendoza M.R., Hsieh Y.-C., Lindbo J., Omarov R.T., Scholthof H.B. (2014). Tombusvirus-based vector systems to permit over-expression of genes or that serve as sensors of antiviral RNA silencing in plants. Virology.

[45] DeMell A., Mendoza M.R., Scholthof H.B., Bayer E. (2023). A tomato bushy stunt virus–based vector for simultaneous editing and sensing to survey the host antiviral RNA silencing machinery. PNAS Nexus.

[46] Gao S.-J., Damaj M.B., Park J.-W., Beyene G., Buenrostro-Nava M.T., Molina J., Wang X., Ciomperlik J.J., Manabayeva S.A., Alvarado V.Y. (2013). Enhanced Transgene Expression in Sugarcane by Co-Expression of Virus-Encoded RNA Silencing Suppressors. PLoS ONE.

[47] Mendoza M.R., Payne A.N., Castillo S., Crocker M., Shaw B.D., Scholthof H.B. (2017). Expression of Separate Proteins in the Same Plant Leaves and Cells Using Two Independent Virus-Based Gene Vectors. Front. Plant Sci..

[48] Sainsbury F., Lomonossoff G.P. (2014). Transient expressions of synthetic biology in plants. Curr. Opin. Plant Biol..

[49] Garabagi F., Gilbert E., Loos A., McLean M.D., Hall J.C. (2012). Utility of the P19 suppressor of gene-silencing protein for production of therapeutic antibodies in *Nicotiana* expression hosts. Plant Biotechnol. J..

[50] Jay F., Brioudes F., Voinnet O. (2022). A contemporary reassessment of the enhanced transient expression system based on the tombusviral silencing suppressor protein P19. Plant J..

[51] Kumar S., Ochoa W., Singh P., Hsu C., Schneemann A., Manchester M., Olson M., Reddy V. (2009). Tomato bushy stunt virus (TBSV), a versatile platform for polyvalent display of antigenic epitopes and vaccine design. Virology.

[52] Grasso S., Lico C., Imperatori F., Santi L. (2012). A plant derived multifunctional tool for nanobiotechnology based on Tomato bushy stunt virus. Transgenic Res..

[53] Joelson T., Morris T.J., Strandberg B., Tomenius K., Akerblom L., Oxelfelt P. (1997). Presentation of a foreign peptide on the surface of tomato bushy stunt virus. J. Gen. Virol..

[54] Blandino A., Lico C., Baschieri S., Barberini L., Cirotto C., Blasi P., Santi L. (2015). In vitro and in vivo toxicity evaluation of plant virus nanocarriers. Colloids Surf. B Biointerfaces.

[55] Lico C., Giardullo P., Mancuso M., Benvenuto E., Santi L., Baschieri S. (2016). A biodistribution study of two differently shaped plant virus nanoparticles reveals new peculiar traits. Colloids Surf. B Biointerfaces.

[56] Lico C., Tanno B., Marchetti L., Novelli F., Giardullo P., Arcangeli C., Pazzaglia S., Podda M.S., Santi L., Bernini R. (2021). Tomato Bushy Stunt Virus Nanoparticles as a Platform for Drug Delivery to Shh-Dependent Medulloblastoma. Int. J. Mol. Sci..

[57] Zampieri R., Brozzetti A., Pericolini E., Bartoloni E., Gabrielli E., Roselletti E., Lomonosoff G., Meshcheriakova Y., Santi L., Imperatori F. (2020). Prevention and treatment of autoimmune diseases with plant virus nanoparticles. Sci. Adv..

[58] He T., Zobeley C., Braun M., Boonrod K., Müller-Renno C., Krczal G., Ziegler C. (2025). Layer-by-Layer Assembly of Plant Viruses Utilizing Specific Binding. Phys. Status Solidi A.

[59] Hsu C., Singh P., Ochoa W., Manayani D.J., Manchester M., Schneemann A., Reddy V.S. (2006). Characterization of polymorphism displayed by the coat protein mutants of tomato bushy stunt virus. Virology.

[60] Cao N., Li Y., Zhang H., Liu X., Liu S., Lu M., Hu Z., Tian L., Li X., Qian P. (2024). A nanoparticle vaccine based on the VP121–26 and VP2 structural proteins of Senecavirus A induces robust protective immune responses. Vet. Microbiol..

[61] Marchetti L., Simon-Gracia L., Lico C., Mancuso M., Baschieri S., Santi L., Teesalu T. (2023). Targeting of Tomato Bushy Stunt Virus with a Genetically Fused C-End Rule Peptide. Nanomaterials.

[62] Marchetti L., Novelli F., Tanno B., Leonardi S., Hizam V.M., Arcangeli C., Santi L., Baschieri S., Lico C., Mancuso M. (2023). Peptide-Functionalized and Drug-Loaded Tomato Bushy Stunt Virus Nanoparticles Counteract Tumor Growth in a Mouse Model of Shh-Dependent Medulloblastoma. Int. J. Mol. Sci..

[63] Müller-Renno C., Remmel D., Braun M., Boonrod K., Krczal G., Ziegler C. (2021). Producing Plant Virus Patterns with Defined 2D Structure. Phys. Status Solidi A.

[64] Rink V., Braun M., Boonrod K., Müller-Renno C., Krczal G., Ziegler C. (2016). Self-assembly of tomato bushy stunt viruses on silicon under the influence of the drop shape, drop volume and the virus concentration. Phys. Status Solidi C.

[65] Lüders A., Müller C., Boonrod K., Krczal G., Ziegler C. (2012). Tomato bushy stunt viruses (TBSV) in nanotechnology investigated by scanning force and scanning electron microscopy. Colloids Surf. B Biointerfaces.

[66] Rink V., Müller-Renno C., Ziegler C., Braun M., Boonrod K., Krczal G. (2017). Electrostatic conditions define the 2D self-assembly of tomato bushy stunt viruses on solid surfaces. Biointerphases.

[67] Müller-Renno C., Rink V., Ani M., Braun M., Boonrod K., Krczal G., Ziegler C. (2020). Bottom-up assembly of a bilayer structure of icosahedral viral nanoparticles. Biointerphases.

[68] He T., Braun M., Boonrod K., Müller-Renno C., Krczal G., Ziegler C. (2024). 2D-Ordered Layers of Tomato Bushy Stunt Virus via Specific Binding. Phys. Status Solidi A.

[69] Zhang X., Ding X., Li Z., Wang S. (2020). Development of Tomato bushy stunt virus-based vectors for fusion and non-fusion expression of heterologous proteins in an alternative host Nicotiana excelsiana. Appl. Microbiol. Biotechnol..

[70] Tleukulova Z., Stamgaliyeva Z., Dildabek A., Mukiyanova G., Omarov R. (2021). Purification of Tomato Bushy Stunt Virus Particles by One-Step Hydroxyapatite Column Chromatography. Eurasian Chem. J..

